# Patterns of stress response to foreign eggs by a rejecter host of an obligate avian brood parasite

**DOI:** 10.1002/ece3.9691

**Published:** 2023-01-18

**Authors:** Mikus Abolins‐Abols, Mark Peterson, Brett Studer, Mattison Hale, Daniel Hanley, George Bentley, Mark E. Hauber

**Affiliations:** ^1^ Department of Biology University of Louisville Louisville Kentucky USA; ^2^ Life‐Science Innovations Willmar Minnesota USA; ^3^ Department of Evolution, Ecology, and Behavior, School of Integrative Biology University of Illinois at Urbana‐Champaign Urbana Illinois USA; ^4^ Department of Biology George Mason University Fairfax Virginia USA; ^5^ Department of Integrative Biology University of California, Berkeley Berkeley California USA

**Keywords:** coevolution, egg rejection, host–parasite interactions, HPA axis

## Abstract

One of the most effective defenses of avian hosts against obligate brood parasites is the ejection of parasitic eggs from the nests. Despite the clear fitness benefits of this behavior, individuals within so‐called “egg‐rejecter” host species still show substantial variation in their propensity to eliminate foreign eggs from the nest. We argue that this variation can be further understood by studying the physiological mechanisms of host responses to brood parasitic egg stimuli: independent lines of research increasingly support the hypothesis that stress‐related physiological response to parasitic eggs may trigger egg rejection. The “stress‐mediated egg rejection” hypothesis requires that hosts activate the stress‐response when responding to parasitic egg stimuli. We tested this prediction by asking whether hosts showed differential stress response when exposed to host‐like (mimetic) or parasite‐like (non‐mimetic) eggs. We experimentally parasitized incubating American robins *Turdus migratorius*, a robust egg‐rejecter host to obligate brood parasitic brown‐headed cowbirds *Molothrus ater,* with mimetic or non‐mimetic model eggs. To assess the stress response, we measured the heart rate in incubating females immediately after experimental parasitism. We also measured plasma corticosterone and, in a subset of birds, used RNA‐sequencing to analyze the expression of proopiomelanocortin (POMC), a precursor of adrenocorticotropic hormone, 2 h after experimental parasitism. We found that egg type had no effect on heart rate. Two hours following experimental parasitism, plasma corticosterone did not differ between the differently‐colored model egg treatments or between rejecter and accepter females within the non‐mimetic treatment. However, females exposed to non‐mimetic eggs showed an upregulation of POMC gene expression (before FDR correction) in the pituitary compared with females treated with mimetic eggs. Our findings suggest that in an egg‐rejecter host species, non‐mimetic parasitic eggs may increase the activity of the stress‐related hypothalamic–pituitary–adrenal axis compared with mimetic eggs, although the temporal dynamics of this response are not yet understood.

## Graphical abstract

Stress‐related signaling has been shown to mediate anti‐parasitic behaviors in avian brood parasite hosts. Here we tested if brood parasite‐like model eggs activated the hypothalamic–pituitary–adrenal (HPA) axis and autonomic nervous system compared with host‐like model eggs. We show that, compared with experimental parasitism with mimetic eggs, parasitism with nonmimetic eggs does not affect blood corticosterone or heart rate, but that exposure to parasitic eggs may increase the expression of POMC gene, a precursor to ACTH, a central hormone in the HPA axis.

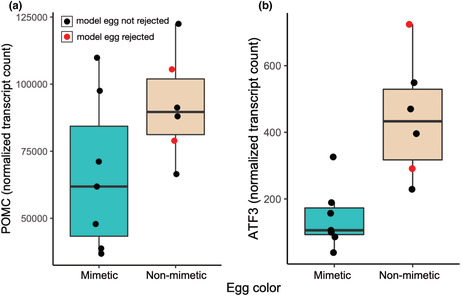

## 1 INTRODUCTION

Obligate avian brood parasites lay their eggs in the nests of heterospecific hosts, imposing substantial fitness costs: parasitized host broods typically experience a lower hatching rate (Hauber, 2003a), higher host chick mortality (Hauber, 2003b), and/or delay in the foster parents' future reproduction (Mark & Rubenstein, 2013). In turn, many host species have evolved behavioral defenses against parasitic eggs. Among the most effective and common host defenses is the ejection of the foreign egg from the nest before it hatches (Davies & Brooke, 1988; Moksnes et al., 1991; Rothstein, 1982). While we know much about the behavioral and sensory ecology and evolution of egg ejection, as well as other forms of parasitic egg rejection (e.g., nest abandonment, the burial of parasitic eggs; Davies & Brooke, 1988; Feeney et al., 2014; Soler, 2014, 2017), the proximate basis of this host defense is still poorly understood (Abolins‐Abols & Hauber, 2018; Ruiz‐Raya, 2021). A mechanistic understanding of a phenotype can offer a unique perspective into the evolution and ecology of organisms (Ketterson et al., 2009; Rosvall, 2013). In particular, understanding the physiological basis of egg rejection may explain one of the most puzzling observations in host–parasite ecology, which is that the propensity to reject parasitic eggs is highly variable both among species (Krüger, 2007; Stokke et al., 2005) and between individuals of the same species (Luro & Hauber, 2017).

While variation in egg rejection across species may be explained by phylogenetic inertia and other evolutionary constraints (Medina & Langmore, 2016; Rothstein, 1975), most of these hypotheses cannot predict why the likelihood of egg rejection varies at the intra‐specific level between populations of individual species (Briskie et al., 1992; Davies & Brooke, 1989; Soler & Møller, 1990), among individuals (Grim et al., 2014; Hauber, Abolins‐Abols, et al., 2020; Hauber, Kim, et al., 2020) and within individuals over time (Ruiz‐Raya & Soler, 2017, 2020). Intraspecific variation in egg rejection is particularly puzzling because this behavior has an obvious fitness benefit and typically incurs comparatively low costs (e.g., the possible rejection of own eggs; Lotem et al., 1995; Ruiz‐Raya & Soler, 2017). Some of the intraspecific variation in egg rejection can be explained by shifts in the sensory‐perceptual environment of the nest (Honza et al., 2011; Rutledge et al., 2021), detecting of an adult brood parasite near the nest (Davies & Brooke, 1988; Moksnes & Røskaft, 1989), or previous experience with parasitic eggs (Hauber et al., 2006). Variation in egg rejection may also be linked to the host life‐history stage (Ruiz‐Raya & Soler, 2017; Zhang et al., 2021). Many of these hypotheses treat egg rejection in isolation from the rest of the phenotype. However, components of egg rejection behavior may share underlying endocrine mechanisms with related behaviors (e.g., aggression, maternal behavior, nest defense; Abolins‐Abols & Hauber, 2018), suggesting that we need to study egg rejection within the rich context of other physiological and behavioral responses of the individual to its environment (Ruiz‐Raya, 2021; Ruiz‐Raya & Soler, 2020).

One of the recently emerged hypotheses about the mechanistic basis of egg rejection suggests that it is mediated by the endocrine stress response (Abolins‐Abols & Hauber, 2018, 2020a; Ruiz‐Raya et al., 2018). For example, Ruiz‐Raya et al. (2018) showed that parasitism with non‐mimetic model eggs elevated baseline corticosterone levels in European blackbird (*Turdus merula*) females. By experimentally suppressing glucocorticoid synthesis, Abolins‐Abols and Hauber (2020a) demonstrated that corticosterone also mediates egg rejection in the congeneric American robin (*T. migratorius*; hereafter “robin”). Specifically, female robins with suppressed glucocorticoid synthesis were less likely to reject non‐mimetic model eggs. In turn, non‐invasively supplemented circulating corticosterone levels caused robins to reject non‐mimetic eggs more frequently (Turner et al., 2022). Combined, these findings imply that brood parasitic egg stimuli may induce a stress response which then modulates the egg rejection decisions and behaviors. Alternatively, the stress response may prime the host physiologically and cognitively to recognize and reject the foreign egg, instead of directly inducing the egg rejection response.

The emerging findings that steroid hormones play a role in response to parasitic eggs in hosts (see also Hahn et al., 2017; Hauber, Abolins‐Abols, et al., 2020) is important not only from a mechanistic perspective but also from an evolutionary one. Glucocorticoids are hormones that impact multiple aspects of the phenotype, including stress response, metabolism, and immune response (MacDougall‐Shackleton et al., 2019; Williams, 2008). Selection on any of these or other glucocorticoid‐mediated aspects of the phenotype may cause population‐ or individual‐level variation in glucocorticoid levels (Ketterson & Nolan, 1999), thus also driving variation in egg rejection. In turn, the glucocorticoid‐mediated response to brood‐parasitic stimuli may drive variation in other aspects of the phenotype. For example, stress‐induced corticosterone secretion can suppress maternal investment (Angelier et al., 2009; Angelier & Chastel, 2009). This suggests that glucocorticoid‐mediated responses to parasitic stimuli may induce a critical trade‐off between host defenses and maternal behavior, as suggested by Abolins‐Abols and Hauber (2018).

While the “stress‐mediated egg rejection” hypothesis has been supported by a number of studies (Abolins‐Abols & Hauber, 2020a; Ruiz‐Raya et al., 2018, 2021), its role in driving individual variation in egg rejection is yet to be fully understood. For example, experimental suppression of glucocorticoid results in a lower propensity for egg rejection (Abolins‐Abols & Hauber, 2020a); however, contrary to the predictions of the stress‐mediated egg rejection hypothesis, natural circulating baseline corticosterone in the same species shows a near‐significant negative association with the propensity for egg rejection (Abolins‐Abols & Hauber, 2020b). To understand whether stress response can drive egg rejection decisions in diverse contexts and across varied timeframes, we need to understand the multifaceted physiological responses of hosts to parasitic eggs.

For example, response to stressful stimuli is considerably more complex than a simple read‐out of corticosterone levels. Corticosterone is regulated by adrenocorticotropic hormone (ACTH) secreted from the anterior pituitary (Romero & Butler, 2007), where it is encoded by the proopiomelanocortin (POMC) gene. The POMC peptide is post‐translationally cleaved to produce a variety of signaling peptides, including ACTH. ACTH secretion from the anterior pituitary is regulated by hypothalamic hormones, such as corticotropin releasing hormone (CRH) and arginine vasotocin (Romero, 2006). The stimulation of the pituitary by CRH results in a rapid release of secretory vesicles containing ACTH (Deng et al., 2015; Harno et al., 2018), which, in turn, stimulates rapid glucocorticoid secretion from the adrenal cortex (Romero & Butler, 2007). While it is clear that glucocorticoids play an important role in regulating behavior and physiology, it is important to note that other components of the hypothalamic–pituitary–adrenal (HPA) axis may have glucocorticoid‐independent extra‐adrenal effects on behavior (e.g., ACTH: Brain & Evans, 1977; Gallo‐Payet, 2016; Miller & Ogawa, 1962). Furthermore, the activation of the HPA axis is only one aspect of a multifaceted response to stressful stimuli. Immediate response to stressors is enabled by catecholamine release (the fight‐or‐flight response), which is regulated by the sympathetic branch of the autonomic nervous system (Romero & Gormally, 2019). Among effects on behavior, catecholamine release results in an elevated heart rate (Cyr et al., 2009). Importantly, while heart rate increases in response to stressors, heart rate may be lowered during orienting response (Hauber et al., 2002).

**FIGURE 1:**
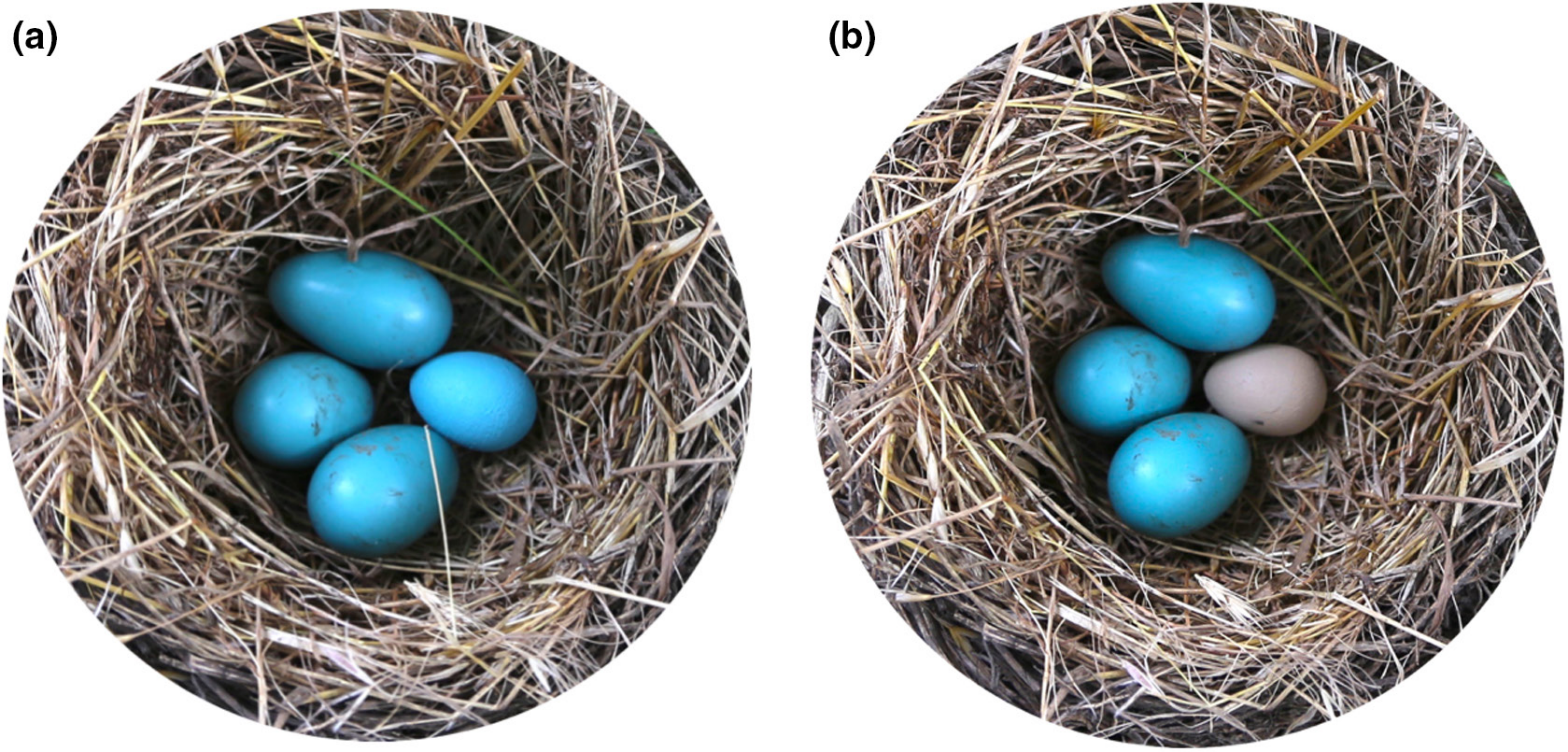
Experimental parasitism treatments in a natural American robin nest: (a) mimetic blue model egg and (b) a non‐mimetic beige model egg

Here we tested the hypothesis that parasitic egg stimuli induce a stress response in a robust egg‐rejecter host, the American robin (Rothstein, 1982). Specifically, robins are one of the handful of rejecter host species that consistently (>80%) remove the eggs of a generalist brood‐parasite, the brown‐headed cowbird (*Molothrus ater*), whereas most of this cowbird's hosts accept (that is, reject fewer than 20% of) foreign eggs or egg models (Winfree, 1999). While most robins (>90%) eject natural or model cowbird eggs from their nests (Luro et al., 2018), some individuals accept natural cowbird eggs and hatch parasitic offspring. Furthermore, variation in egg rejection is repeatable in robins (Luro & Hauber, 2017), making this species a great model system in which to test the mechanisms underlying variation in egg rejection. We experimentally parasitized robin females with mimetic (robin‐like color) or non‐mimetic (cowbird‐like color) eggs (Figure 1) and measured three aspects of host physiology in response to the model eggs: heart rate, corticosterone levels, and POMC expression in the pituitary. We predicted that robin females exposed to the non‐mimetic model eggs would show increased heart rate, greater circulating corticosterone, and higher POMC expression compared with females receiving mimetic eggs. Alternatively, if females show orienting response toward non‐mimetic eggs, we predicted decreased heart rate in response to non‐mimetic compared with mimetic eggs.

## 2 MATERIALS AND METHODS

We studied American robin females breeding in a tree farm near Urbana, IL, USA (lat: 40.128N; long: −88.105E) from April–July in 2018 and 2019. We focused only on female robins as it is the sex responsible for egg rejections in this species (Hauber et al., 2019). We surveyed the area every 3 days to detect incubating females. The stage of incubation was unknown for most subjects as in this study we prioritized maximizing sample sizes over following nest progress, and we therefore did not include it in our analyses.

### 2.1 Experimental parasitism treatment

To assess whether parasitic eggs induce a stress response in American robin females, we experimentally parasitized their nests during incubation with either a mimetic or non‐mimetic model egg (Figure 1). We used 3D printed white nylon eggs, sourced from Shapeways.com (model ID “cow bird”), which were modeled after a natural cowbird egg and are similar in shape, size, and weight to it (Igic et al., 2015). These eggs were painted either immaculate light blue (mimetic treatment, resembling natural robin eggshell's color) or immaculate beige (non‐mimetic treatment, resembling the cowbird eggshell's color) following published protocols (Canniff et al., 2018; Hauber et al., 2019). We placed one model egg in the nest of the incubating female, without removing any host eggs: prior studies showed that the removal of own eggs in *Turdus* thrushes did not affect their responses to experimental parasitism (Grim et al., 2011). Variation in natural or artificial olfactory cues also does not induce egg rejection in American robins (Hauber, 2020).

### 2.2 Capture

To assess circulating corticosterone (*n* = 43) and pituitary gene expression for a subset of subjects (*n* = 13), we captured experimentally parasitized females 2 h after the addition of a model egg. The 2 h time point was chosen because plasma corticosterone in birds typically increases rapidly in the first 30 min to an hour following an exposure to an acute stressor (such as handling or nest disturbance), after which plasma corticosterone titer remains near its maximum if the stressor persists (Abolins‐Abols et al., 2016; Breuner et al., 2006; Meddle et al., 2003; Romero et al., 1998). For logistical reasons, we were not able to remain near the nest to determine the return time of each female, but in this population, females typically arrive back to their nests on average 12 min after being flushed (pers. obs. *n* = 31, standard deviation (SD) = 7.37, range = 2–28). Most of the females (86%: 38 out of 43) were flushed from their nests prior the insertion of the model egg (the proportion of flushed females did not differ between the treatments (Fisher's exact test, *p* = 1.0)). The 2‐h time window therefore provided most of the females with an opportunity for a prolonged interaction with the model egg before capture, allowing any corticosterone increase in response to the model eggs to reach its maximum.

Previous research also showed that most female robins' decisions to reject or accept a model egg are not immediate but occur on the timescale of hours rather than minutes (Hauber et al., 2019; Scharf et al., 2019). Importantly, the only other study we are aware of in *Turdus* thrushes investigating the effect of experimental parasitism on plasma corticosterone detected elevated corticosterone 24+ hours after the addition of the model egg (Ruiz‐Raya et al., 2018). However, sampling robins at 24 h following the experimental parasitism without tethering the model eggs would have meant that nearly all the females would have rejected the non‐mimetic parasitic eggs (e.g., Hauber, Hoover, et al., 2020) and that they would therefore have been unstimulated for several hours before capture. The 2‐h time window thus allowed us to catch females during or closely following stimulation by model eggs, and, according to our estimate, allowed sufficient time for any changes in corticosterone in most females reach or remain near peak levels.

We captured females with a 6 m mist net, arranged in a V‐shape around the nest tree. The poles and a rolled‐up closed mist net were set up immediately before the addition of the model eggs. Two hours after the initial net setup and the addition of the model egg, we unfurled the mist net and captured the female either by flushing it into the net or passively capturing it as it attempted to land on the nest. Setting up the net prior to experimental parasitism enabled rapid set up and capture following the 2‐h mark (*n* = 42, mean capture time = 15.33 min, min = 0, max = 36, SD = 9.19) and allowed us to minimize any changes to corticosterone levels due to nest disturbance. Importantly, we found no association between baseline plasma corticosterone concentration (see below for corticosterone assay methods) and the time it took to catch the female (Pearson's *r* = −.16, df = 40, *p* = .30) or whether the female was flushed from the nest (two‐tailed *t*‐test, *t* = 0.15, df = 4.64, *p* = .88). Therefore, we did not account for these metrics in the statistical models testing the effect of egg type on corticosterone levels (see section below). At the time of capture, we also noted whether the model egg was ejected from the nest or still present (“accepted”).

### 2.3 Corticosterone sampling and measurement

We sampled 75 μl blood from each subject's brachial vein within 3 min of capture (mean = 157.9 s, min = 85, max = 216, SD = 30.877; for two birds their blood collection ended after the 3 min mark). The time to end blood collection was not correlated with natural log‐transformed baseline corticosterone levels (Pearson's *r* = .03, df = 41, *p* = .87) and we did not account for it in the statistical models.

Blood was kept on ice until centrifugation 2–8 h later at +4°C for 10 min at 5740 *g*. Following centrifugation, blood plasma was removed and stored at −80°C until analysis. Plasma corticosterone was measured using enzyme immunoassay (Cayman Chemical, catalogue number 501320) which has been validated and optimized for American robins (Abolins‐Abols & Hauber, 2020b). We extracted the non‐polar components of plasma using diethyl ether extraction, described in Abolins‐Abols & Hauber (2020b). Briefly, 10 μl plasma was suspended in 200 μl ultrapure water and mixed with 1 ml diethyl ether. After passive phase separation, the mixture was flash‐frozen, and the ether phase was decanted into a fresh vial. Ether was then evaporated using nitrogen gas at 40°C. Ether was added to the aqueous portion and decanted two more times. The extract was suspended in 600 μl assay buffer overnight at 4°C. The concentration of corticosterone in the extract was measured according to the manufacturer's instructions. Each extracted sample was analyzed in triplicate. We measured absorbance at 405 nm using Biotek 800TS plate reader and analyzed the data using an 8‐point logistic curve using the Cayman Chemical analysis spreadsheet. The average intraplate coefficient of variation (CV) was 5.78%, while inter‐plate CV was 6.19%; we therefore averaged values from the three replicates for each sample and standardized concentrations across places using the mean concentration of a standard control sample, for use in the statistical models.

### 2.4 Gene expression analysis

Immediately following blood collection, a subset (*n* = 13) of females was euthanized using isoflurane overdose, followed by rapid decapitation. The sample size for gene expression analyses was small to limit the number of euthanized wild songbirds. The brain was dissected and reserved for another study. The skull with the embedded pituitary gland was immediately flash‐frozen on pulverized dry ice and transferred to a −80°C freezer within 6 h. Pituitaries were then rapidly dissected from frozen skulls and returned to −80°C storage.

For RNA‐extraction, the tissue was suspended in 500 μl Tri‐Reagent (Molecular Research Center) and homogenized. RNA was then extracted using the manufacturer's protocols, treated with DNase I (New England Biolabs) and purified using QIAGEN RNeasy mini kit.

RNAseq libraries were constructed at the DNA Services laboratory of the Roy J. Carver Biotechnology Center at the University of Illinois at Urbana‐Champaign using the TruSeq Stranded RNA Sample Preparation Kit (Illumina). Briefly, the total RNA was quantitated by Qubit (Life Technologies), then PolyA+ RNA was selected from 1 μg of total RNA per sample. PolyA+ RNA was fragmented for 4 min at 94°C, then first‐strand cDNA was synthesized with a random hexamer and SuperScript II (Life Technologies). Double stranded DNA was blunt‐ended, 3′‐end A‐tailed and ligated to unique dual‐indexed adaptors. The adaptor‐ligated double‐stranded cDNA was amplified by PCR for 10 cycles with the Kapa HiFi polymerase (Kapa Biosystems). The final libraries were quantitated on Qubit and the average size determined on the AATI Fragment Analyzer (Advanced Analytics) and diluted to 5 nM final concentration. The 5 nM dilution was further quantitated by qPCR on a BioRad CFX Connect Real‐Time System (Bio‐Rad Laboratories, Inc.).

The final stranded RNASeq library pool consisting of 13 libraries was sequenced on 1 lane of an Illumina NovaSeq 6000 SP flowcell as paired‐reads with 150 nt length. The run generated .bcl files which were converted into adaptor‐trimmed demultiplexed fastq files using bcl2fastq v2.20 Conversion Software (Illumina).

### 2.5 Heart rate analysis

**FIGURE A1:**
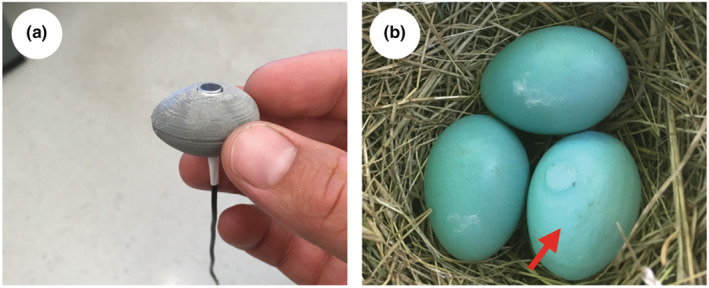
Microphone‐fitted model egg for heart rate recording. (a) model egg before it is covered by a balloon and painted. The microphone is positioned on the egg equator; (b) finished and painted microphone‐fitted egg (indicated by the red arrow) placed in a natural American robin nest. The egg is positioned so that the microphone (covered by a balloon) makes contact with the brood patch of an incubating female.

In 2018, we exposed an additional set of robin females (*n* = 14) to the same binary model egg treatments and measured their heart rate during incubation immediately following the experimental parasitism. We measured heart rate by adopting an approach from Arnold et al. (2011) where a microphone‐fitted model egg (“microphone egg” hereafter) is used to record audio signatures of heartbeats in incubating females through contact with the featherless brood patch. To make a robin egg‐like microphone egg, we 3D‐printed a custom model eggshell resembling American robin eggs in size and shape using MakerBot Replicator Mini+3D printer (MakerBot). The model shell consisted of two halves, which connected along the long axis of symmetry. The two halves each had an opening on the egg equator—one half had an opening on for a 6 mm microphone, the other had an opening for a headphone cord (Figure A1a in Appendix 1). After printing, each microphone egg's surface was smoothed using sandpaper. Each egg was then fitted with a unidirectional microphone (PUM‐3546L‐R, PUI Audio Inc.). The microphone was soldered to a headphone cable and inserted into the model egg (“top” side of the egg), with the headphone cable leaving the model egg on the opposite (“bottom”) side of the egg (Figure A1a in Appendix 1). A white rubber balloon (Walmart) was then stretched over the egg and tied with a fishing line around the headphone cable. The microphone egg was then painted in mimetic robin‐blue following the methods outlined above.

To insert the microphone egg in the nest, we first removed all the robin eggs to prevent damage to them. We then poked a small hole in the mud‐lined bottom of the robin's nest cup through which we passed the headphone cable, leaving the model egg resting on the bottom of the nest with the microphone side facing up (Figure A1b in Appendix 1). The robin eggs were then returned to the nest, typically within 1 min of their removal. One robin egg was removed to prevent host clutch to change by more than one egg following the experimental treatment (see below). The headphone cord was then connected to a digital sound recorder (Olympus Digital Voice Recorded WS‐852, Olympus).

**FIGURE A2:**
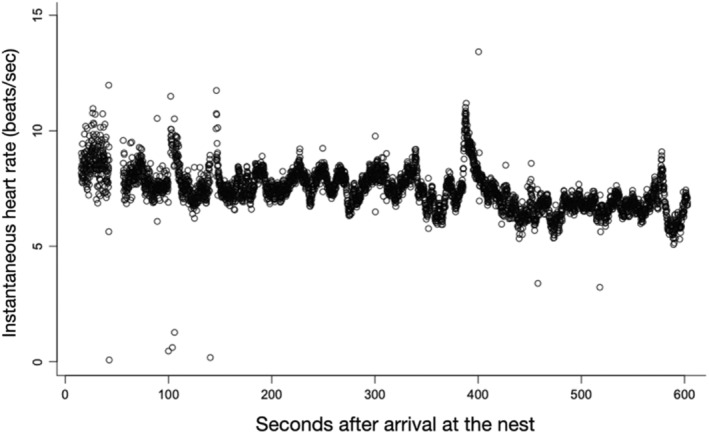
Example heart rate trace from an incubating female after arrival at the nest. Each dot indicates instantaneous heart rate for each heartbeat. Heartbeats were scored by hand and the instantaneous heart rate calculated using formula 1/(time between two successive heart beats).

We first placed the microphone egg into the focal nest a day before the experimental model egg addition to minimize the disturbance at the nest. We recorded 2 h of heartbeat audio signatures to check that the model egg was contacting the brood patch and recording audio signatures of heart beats (Figure A2 in Appendix 1). One day following the insertion of the microphone egg, we returned to the nest and inserted either mimetic blue or non‐mimetic beige cowbird‐sized solid nylon model eggs (as described above under “Experimental parasitism treatment”) in the focal nest and started to record the heart rate again. We returned to the nest 2 h following the experimental parasitism, removed the experimental model egg and replaced it with the opposite treatment. For example, in nests where a female was experimentally parasitized with a non‐mimetic beige egg, the beige egg was removed and a mimetic blue egg was added in its place (and vice versa). Each female was therefore sequentially exposed to both mimetic and non‐mimetic treatments, but the order of the treatments was randomized among subjects. Two hours after the addition of the second experimental egg we removed both the experimental and the microphone egg.

We used loud rustling sounds to determine the arrival of the female at the nest cup. We then filtered the heart rate audio recordings to remove sounds above 1 KHz frequencies using Adobe Audition (Adobe). We then manually scored the heartbeats using R programming environment using seewave (Sueur et al., 2008) and tuneR (Ligges et al., 2018) packages following published protocols (Sueur, 2018). Heartbeats were then transformed into instantaneous heart rate (beats/sec) using the following formula: 1/*i*, where *i* is the interval (s) between two successive heart beats. Unlike most endocrine responses, heart rate can change in a matter of seconds and can reflect instantaneous responses to stimuli (Wascher, 2021) therefore we limited our analyses to the first 10 min following the arrival of the focal female.

### 2.6 Statistical analyses

All analyses were conducted using R programming environment (R Core Team, 2017). The effect of egg type (mimetic vs. non‐mimetic) on corticosterone levels was analyzed using a linear model. Corticosterone levels were natural log‐transformed to conform to the expectations of normal distribution (log‐transformed corticosterone: Shapiro–Wilk *W* = 0.966, *p* = .238). In addition to the egg type, we included the date of the manipulation as a fixed factor in the analyses, because maternal hormone levels often show strong seasonality (Hauber, Abolins‐Abols, et al., 2020; Jawor et al., 2006; Tyrrell & Cree, 1998) and in this study corticosterone showed a significant decrease over the breeding season (see below). We then used a linear model with date as a covariate to test if corticosterone levels differed between females who rejected or accepted a non‐mimetic egg at the 2‐h mark.

For RNAseq analysis, all paired‐end reads were mapped to the Swainson's thrush *Catharus ustulatus* genome (Accession GCF_009819885.1) as it was the closest available full genome assembly at the time of this analysis. Paired‐end mapping was completed using rsem (Li & Dewey, 2011) and bowtie 1.0.0 (Langmead et al., 2009) with default parameters. To exclude unexpressed genes and genes not present in robins, only genes with at least 10 identified read counts in over one‐third of samples were included for analysis. Differential expression was analyzed using DESeq (Anders & Huber, 2010) for differences between treatments and differences between individuals who rejected and those that did not reject the mimic egg. For all analyses, the threshold for significance was set at a false‐discovery‐rate of 5%.

We calculated instantaneous heart rate each minute, for the first 10 min, after the bird arrived at the nest (first audible detection). The average instantaneous heart rates over this 10‐min period were then log‐transformed to satisfy assumptions of normality (log‐minute averages: Shapiro–Wilk *W* = 0.992, *p*‐value = .058). We then used linear mixed models using R package nlme (Pinheiro et al., 2015) to analyze the effect of model egg color on average heart rate, adding time since arrival, treatment date, and treatment order as covariates, and bird ID as a random factor. Time since arrival was included because robin heart rate was typically high immediately after the arrival at the nest (presumably due to the metabolic demands of flight) following which the heart rate rapidly decreased. To test if individuals differed in the rate of which their heart rate declined following the arrival, we asked if addition or random slope terms to the model resulted in a better model fit. However, random slope models had a higher AIC, therefore the final models did not include the random slope term. Only one female rejected the non‐mimetic beige egg within the 10 min heart rate recording window and we, therefore, did not include the rejection as a covariate in the model. However, we ran a separate model asking if females that rejected the non‐mimetic beige egg within 2 h had a higher heart rate in response to the beige eggs compared with females that did not reject these eggs within this time period.

## 3 RESULTS

**FIGURE 2:**
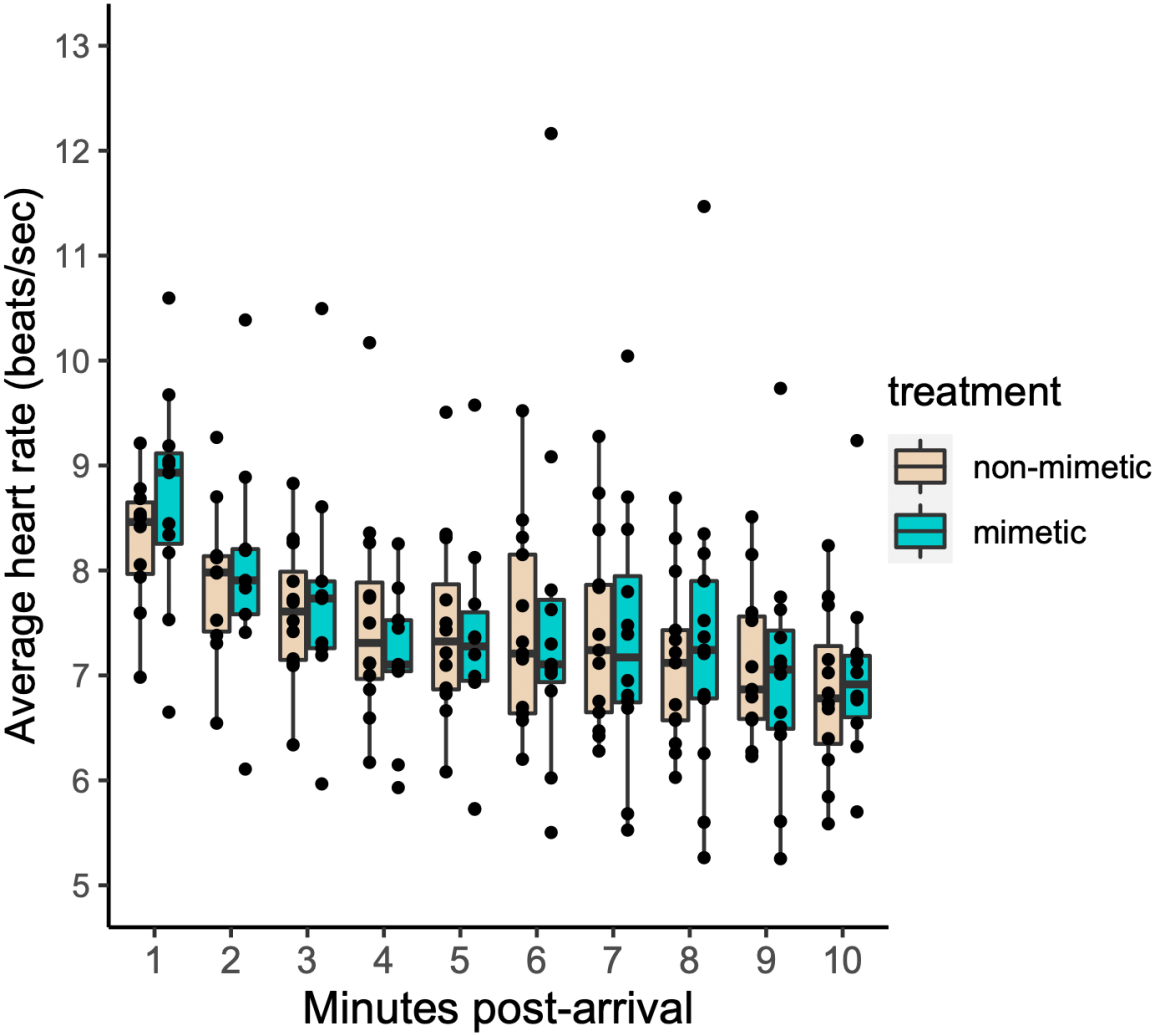
The effect of model egg treatment on heart rate. Heart rate following the addition of non‐mimetic beige eggs did not differ from heart rate following the addition of mimetic blue egg in a separate set of experimentally parasitized females (*n* = 21). Black dots show time‐specific average heart rate for individual females.

Heart rate showed a significant decrease with time after arrival at the nest (LMM, estimate = −0.020, df = 208, *t* = −10.530, *p* < .001), but variation in heart rate was not explained by the treatment date (LMM, estimate = 0.002, df = 11, *t* = 0.485, *p* = .637) or treatment order (LMM, estimate = 0.051, df = 11, *t* = 0.682, *p* = .509). In this subset of females, model egg color type had no statistical effect on incubating females' (*n* = 14) heart rate immediately after return to the nest (LMM, estimate = 0.009, df = 208, *t* = 0.824, *p* = .411, Figure 2). Furthermore, initial heart rate did not differ between rejecter and acceptor females during the non‐mimetic egg trials (LMM, estimate = −0.053, df = 9, *t* = −0.875, *p* = .404). Eight out of 14 females (57%) rejected the non‐mimetic egg within 2 h.

**FIGURE 3:**
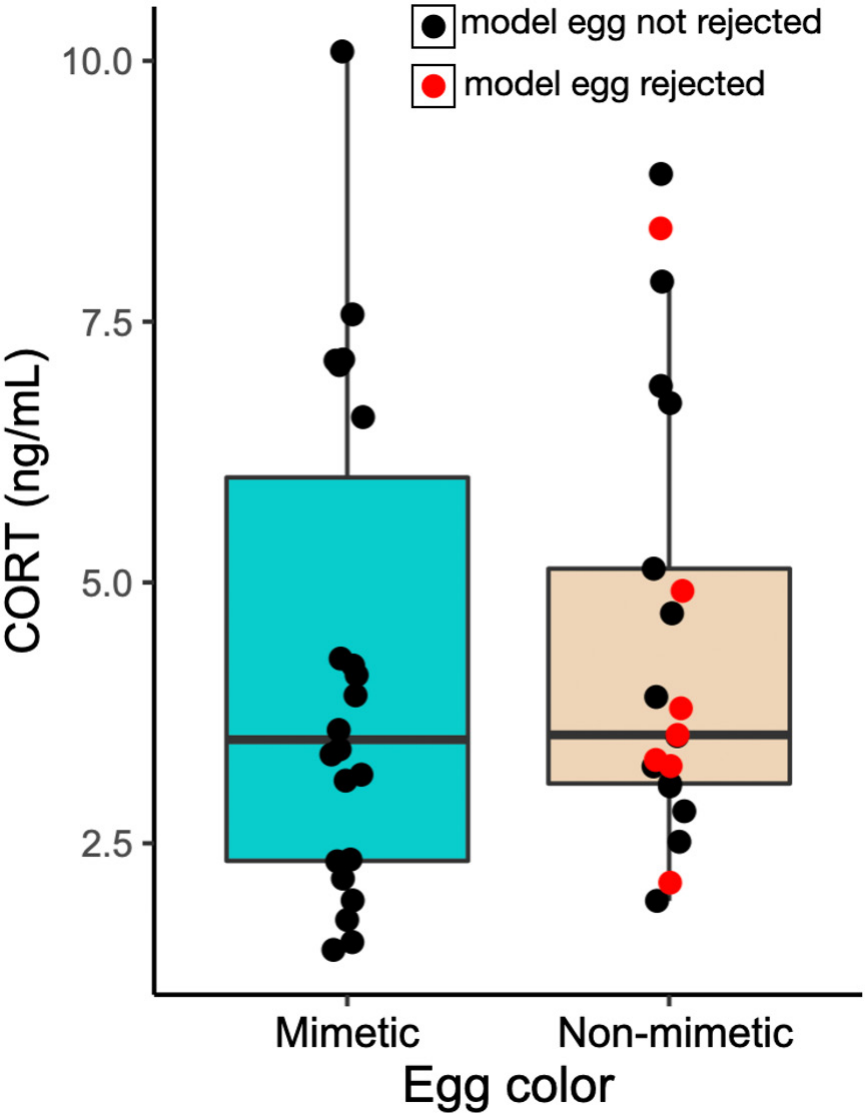
The effect of model egg treatment on corticosterone levels. Baseline corticosterone did not differ between females experimentally parasitized with non‐mimetic beige eggs (*n* = 21) or mimetic blue eggs (*n* = 22). Black dots indicate females that had not rejected the model egg within 2 h, while red dots indicate females that rejected the model egg. Within the non‐mimetic egg treatment, females that rejected and accepted the eggs did not differ in their corticosterone levels.

In the second subset, females exposed to mimetic or non‐mimetic model eggs did not show significant differences in circulating corticosterone concentrations 2 h after experimental parasitism (LM, estimate: −0.118, *t* = −0.913, df = 40, *p* = .367; Figure 3). Plasma corticosterone concentrations decreased across the season (LM, estimate = −0.012, *t* = −4.301, df = 40, *p* < .001). A significantly higher proportion of the non‐mimetic egg eggs were rejected (seven out of 21; 33%) compared with mimetic blue eggs (0 out of 22; 0%) within 2 h of their addition (Fisher's exact test, *p* = .0036). However, plasma corticosterone did not differ between females who rejected or accepted the non‐mimetic egg (LM, estimate: −0.008, df = 18, *t* = −0.044, *p* = .966; Figure 3).

**FIGURE 4:**
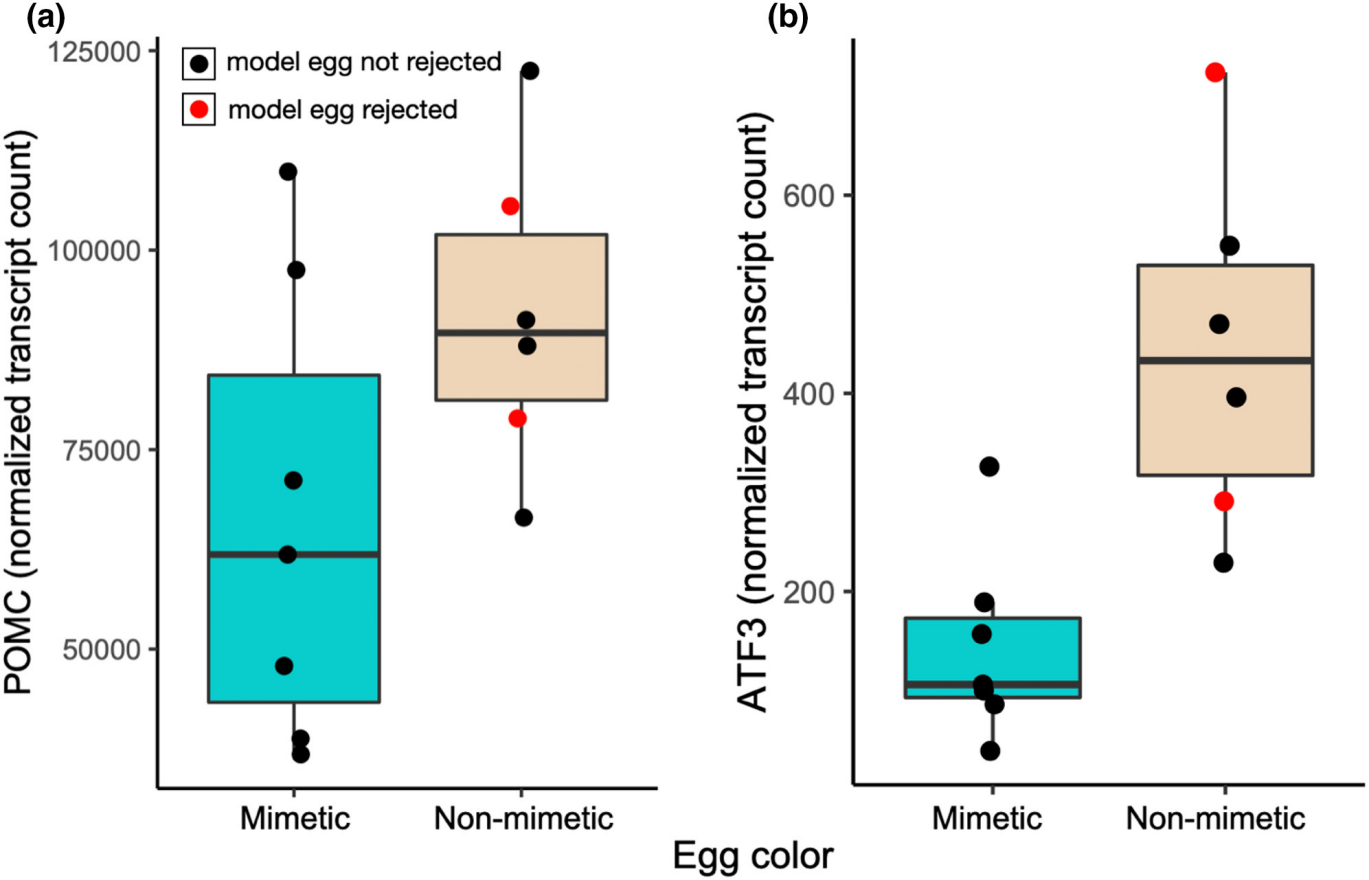
The effect of model egg treatment on (a) POMC and (b) ATF3 expression in the pituitary in a subset of female American robins. Prior to FDR correction, females experimentally parasitized with non‐mimetic beige eggs (*n* = 6) showed higher POMC and ATF3 expression compared with females experimentally parasitized with mimetic blue eggs (*n* = 7). Black dots indicate females that had had not rejected the model egg within 2 h, while red dots indicate females that rejected the model egg. Neither POMC nor ATF3 expression differences were significant after FDR correction.

**FIGURE 5:**
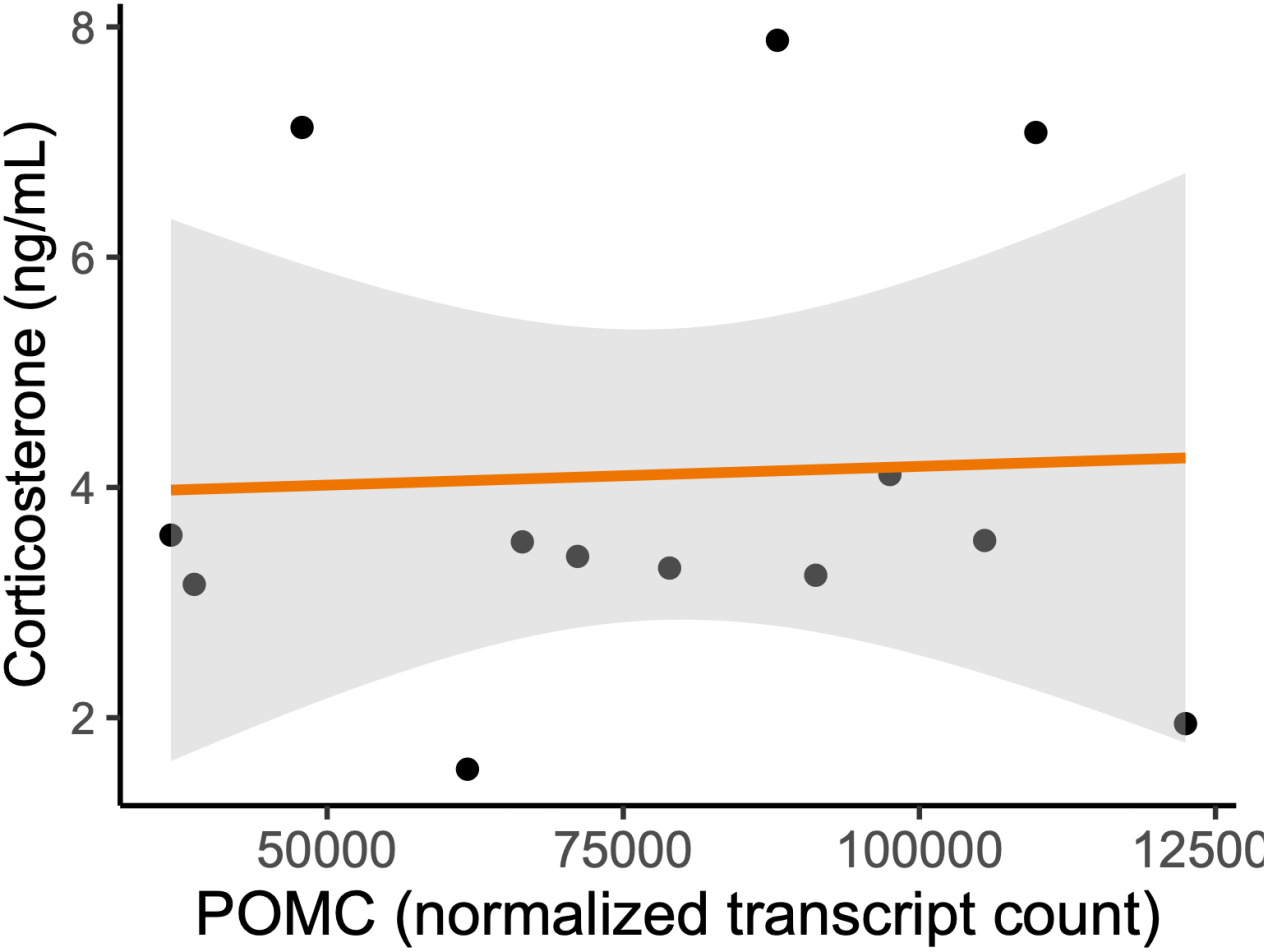
POMC expression in the pituitary was not correlated with plasma corticosterone concentration 2 h following experimental parasitism (*n* = 13) of American robins. Shaded area indicates the 95% confidence interval.

Prior to false discovery rate (FDR) correction, 312 pituitary‐expressed genes were significantly differentially expressed between birds exposed to the mimetic and non‐mimetic egg treatments (Table S1) 2 h following the experimental parasitism. Among these, POMC showed higher expression in pituitary in birds exposed to non‐mimetic model eggs (*n* = 6) compared with birds exposed to mimetic eggs (*n* = 7, fold‐change = 0.719, *p* = .042, Figure 4). One of the most significantly differentially expressed genes prior to FDR correction was ATF3, a transcription factor specifically associated with the stress response (Hai et al., 1999), which was upregulated in the birds exposed to non‐mimetic eggs (fold change = 0.323, *p* < .001). No genes were significantly differentially expressed between the model egg treatments following FDR correction. POMC expression levels and corticosterone concentrations were not correlated with each other (Spearman's *ρ* = 0.055, df = 11, *p* = .863, Figure 5).

## 4 DISCUSSION

We found that experimental nest parasitism with a non‐mimetic egg does not elevate circulating plasma corticosterone compared with parasitism with a mimetic egg in a typically egg‐rejecting host of an obligate avian brood parasite 2 h following the addition of the model eggs. However, at the same time point, the expression of the HPA axis‐relevant POMC gene and the stress‐related transcription factor ATF3 in the pituitary was elevated in birds exposed to the non‐mimetic model egg compared with the individuals exposed to the mimetic egg, although these differences were not significant after false discovery rate correction. FDR limits the type‐I error in datasets with a large number of comparisons. However, given that we were mainly interested in a few HPA‐axis candidate genes in the pituitary, we also report the uncorrected *p*‐value, while still acknowledging the possibility of this result being a false positive. We detected no changes in heart rate responses of incubating robins between our model egg‐color treatments.

Our experiment was designed to ask whether the differential behavioral responses to parasitic eggs of different colors were paralleled by differential physiological responses to these eggs. While our findings cannot inform the question whether the presence or absence of a parasitic egg causes stress responses in robins, our results are consistent with non‐mimetic coloration of brood parasitic eggs causing a delayed, but detectable, increase in HPA axis activity compared with the physiological response to mimetically‐colored eggs. This provides a possible mechanism for the observation that robin females reject non‐mimetic eggs more readily than mimetic eggs. In this study, we measured POMC mRNA levels in the pituitary, not ACTH itself. Although POMC gene expression can be upregulated within 30 min of CRH stimulation (Levin et al., 1989), translation and posttranslational modification of the POMC peptide into ACTH likely take time, as POMC needs to be packaged in vesicles where it is cleaved into daughter peptides (Pritchard & White, 2007). Thus, it is possible that a POMC‐related corticosterone increase occurs after our 2‐h mark. Indeed, we found no association between POMC expression and corticosterone levels at the time of sampling our subjects. Alternatively, it is possible that at 2 h we already missed a rapid elevation of circulating glucocorticoids following exposure to the non‐mimetic model egg or that the *p*‐value based detection of the increased POMC gene expression data is simply a statistical artifact. ATF3, which was upregulated in birds exposed to non‐mimetic eggs, is typically upregulated in cells experiencing physiological stress (Hai et al., 1999), although ATF3 expression can also be induced by psychological restraint stressor (Green et al., 2008). The upregulation of ATF3 in the pituitary in response to a brood‐parasitic egg thus supports the hypothesis that parasitic eggs are perceived as stressful by the hosts, although the role of ATF3 expression in the pituitary is unclear.

In a different study investigating changes in corticosterone in response to parasitic eggs in a congeneric species (European blackbird) an increase in baseline corticosterone was detected 24+ h following experimental parasitism with a non‐mimetic egg relative to no‐treatment control (Ruiz‐Raya et al., 2018). Although their study did not test whether this increase in corticosterone was due to the addition of the egg or the egg being non‐mimetic, one of the interpretations of their results is that distinctive parasitic eggs may cause a slow or delayed increase in HPA activity over multiple hours. Consistent with this interpretation, we found no difference in the heart rate between the non‐mimetic and mimetic egg treatments immediately following the arrival of the female at the nest. Heart rate can serve as a rapid, real‐time indicator of acute stress response (Cyr et al., 2009), and the lack of differences in the heart rate immediately after encountering the egg suggests that parasitic stimuli do not cause an acute stress response.

A low, but long‐term increase in HPA activity may have functional significance in the context of modulating rejection of non‐mimetic eggs, even if it is not statistically significant at a single time point of sampling. For example, Abolins‐Abols and Hauber (2020a) found that long‐term (overnight) suppression of glucocorticoid synthesis increases the acceptance rate of parasitic eggs by American robins. If natural glucocorticoid release in response to parasitic eggs stimulates egg rejection, then minimal but long‐term upregulation of the HPA axis in response to non‐mimetic parasitic egg stimuli is consistent with the timeframe of rejection of parasitic eggs by American robins. For example, >90% of robins reject non‐mimetic beige cowbird‐sized eggs within 5 days (Luro et al., 2018), 2 days (Hauber et al., 2019), and even 1 day (Hauber, Hoover, et al., 2020), but only 33% (seven out of 21) birds rejected the same non‐mimetic egg type within 2 h in this study (also see Scharf et al., 2019). However, individual variation in glucocorticoid levels in this study did not predict egg rejection: circulating corticosterone at our single time‐point of sampling did not differ between females who accepted or rejected the non‐mimetic beige eggs (Figure 3).

The few steroid‐focused endocrine studies on physiological responses to parasitic egg stimuli so far thus paint a complicated picture. On one hand, experimental studies now show that glucocorticoids (Abolins‐Abols & Hauber, 2020a) can mediate egg rejection. On the other hand, the HPA axis may only show weak gradual activation in response to exposure to non‐mimetic brood parasitic eggs and may have no discernible effect on egg rejection. It is possible that egg rejection by brood parasite hosts may be affected only by pronounced changes in the HPA activity (such as those due to experimental manipulation of hormone levels or intense stressors) and not by weak HPA activation in response to brood parasitism. Furthermore, the relationship between stress physiology and egg rejection may be species‐specific. For example, in incubating prothonotary warblers (*Protonotaria citrea*), a non‐rejecter host species, experimental parasitism with either non‐mimetic or mimetic eggs had no effect on glucocorticoid levels relative to non‐parasitized controls (Scharf et al., 2021). On the other hand, in yellow warblers (*Setophaga petechia*), a species that frequently but variably abandons its nest if parasitized by brown‐headed cowbirds, individuals that deserted an experimentally parasitized nest had higher circulating corticosterone levels relative to non‐parasitized controls (Turcotte‐van de Rydt et al., 2022).

Methodologically, especially when studying freely behaving wild animals, we are still limited by our inability to measure the minute‐by minute dynamics in glucocorticoid synthesis and release, as well as our inability to accurately assess long‐term subtle changes in glucocorticoids (and other hormones) that could cause a behavioral change. To integrate our findings into a unified framework, future experiments should characterize the full‐time course and magnitude of HPA axis activation in response to brood parasitism across acceptor and rejector species. Additionally, dose–response studies, such as those inducing small and short‐term changes in hormone levels (e.g., Vitousek et al., 2018) are necessary to test the sensitivity of host behavior to hormones. Finally, future studies must expand beyond the corticosterone paradigm and test the effect of other hormones, such as prolactin, on host behaviors (Abolins‐Abols & Hauber, 2018; Ruiz‐Raya et al., 2021), as well as stress‐response at the cellular level (Ruiz‐Raya et al., 2022). Together, these studies will allow us to more fully understand the significance of stress signaling in the ecology and evolution of host defenses.

## 5 CONCLUSIONS

We tested one of the predictions of the “stress‐mediated egg rejection” hypothesis, which predicts that hosts should mount physiological stress response when they perceive cues of brood parasitism. Specifically, we asked whether hosts showed differential stress response when exposed to mimetic or non‐mimetic eggs. Our results are partially consistent with this prediction: while we show that POMC expression is elevated in birds exposed to non‐mimetic eggs compared with mimetic eggs 2 hours after experimental parasitism, plasma corticosterone levels did not differ between the treatments at this timepoint, and experimental parasitism with non‐mimetic eggs did not affect heart rate. These findings suggest that to understand the applicability and the ecological relevance of stress‐mediated egg rejection, we need to address the diversity and subtlety of the stress‐response and its effects on behavior in egg‐rejecter hosts of brood parasites.

## AUTHOR CONTRIBUTIONS

**Mikus Abolins‐Abols:** Conceptualization (equal); data curation (lead); formal analysis (equal); investigation (lead); methodology (equal); software (equal); visualization (equal); writing – original draft (equal). **Mark Peterson:** Formal analysis (equal); writing – review and editing (equal). **Brett Studer:** Investigation (equal). **Mattison Hale:** Formal analysis (equal); writing – review and editing (equal). **Daniel Hanley:** Conceptualization (equal); formal analysis (equal); software (equal); writing – review and editing (equal). **George Bentley:** Methodology (equal); writing – review and editing (equal). **Mark E. Hauber:** Conceptualization (equal); funding acquisition (lead); project administration (equal); resources (lead); writing – review and editing (equal).

## CONFLICT OF INTEREST

All authors have no conflicts of interest to declare.

## Supporting information


Table S1.

## Data Availability

Data are available through Figshare.com: https://10.6084/m9.figshare.21642062.v1.
