# Supplementary material for: Patterns of stress response to foreign eggs by a rejecter host of an obligate avian brood parasite
Source: Ecol Evol. 2023 Jan 18;13(1):e9691. doi: 10.1002/ece3.9691 (PMC9848814; doi:10.1002/ece3.9691)
Supplement: Supplementary file 1 — Table S1. [file ECE3-13-e9691-s001.zip › Table S1legend.docx]

**Table A1.** Differential gene expression of pituitary genes between birds exposed to the mimetic and non-mimetic egg treatments.

*Link to table*: https://doi.org/10.6084/m9.figshare.21603831
